# Unraveling the Power of Disorder: A Bioinspired Cuttlebone Structure With Superior Strength, Energy Absorption and Isotropy

**DOI:** 10.1002/advs.75605

**Published:** 2026-05-08

**Authors:** Zengqin Shi, Peng Jiang, Xiaoyan Zhu, Suyun Li, Wenqing Wang, Tian Zhao, Rujie He, Ying Li

**Affiliations:** ^1^ Institute of Advanced Structure Technology Beijing Institute of Technology Beijing China; ^2^ Marine Science and Technology Domain Beijing Institute of Technology Zhuhai China; ^3^ School of Continuing and Lifelong Education National University of Singapore Singapore Singapore; ^4^ Institute of Systems Science National University of Singapore Singapore Singapore

**Keywords:** biomimetic, buckling, discrete disordered structure, energy absorption, shear isotropy

## Abstract

Lightweight architected materials with superior mechanical performance and tailored isotropy are of critical importance for advanced engineering applications such as aerospace and protective structures. Inspired by the discrete disordered structure of cuttlebone, this study proposes a “controlled disorder” design strategy. Leveraging micro‐CT to extract structural features, discrete topological equations are employed to tailor the unit cells, which are then fabricated into biomimetic specimens via 3D printing. Experiments and simulations reveal that as the level of disorder increases, the compressive strength and specific energy absorption significantly rise by approximately 41% and 206%, respectively, while the modulus remains remarkably constant. Concurrently, shear anisotropy is markedly reduced, with the ratios of minimum to maximum shear stiffness and shear strength increasing by 1981 % and 45 %, respectively, the latter exceeding 0.9—signalling a dramatic transition toward isotropy. Increasing the number of unit cells further promotes isotropy. Notably, the fully disordered specimens outperform existing high‐performance benchmarks in both normalized specific strength and specific energy absorption. Overall, this work uncovers the strengthening and isotropizing mechanisms in biomimetic discrete disordered structures, providing a novel perspective and a robust design paradigm for the development of next‐generation high‐performance mechanical metamaterials.

## Graphical abstract

Cuttlebone‐inspired architected metamaterials introduce controlled structural disorder to simultaneously enhance strength, energy absorption, and compressive stability. Tunable disorder suppresses shear anisotropy, outperforms periodic lattices, and establishes discrete randomness as a robust route to high‐performance lightweight mechanical metamaterials.

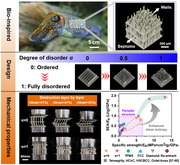

## 1 Introduction

Mechanical metamaterials have revolutionized the landscape of materials science by decoupling properties that are traditionally coupled in bulk solids, such as strength and density or stiffness and compressibility [1, 2]. Unlike conventional materials whose properties derive primarily from their chemical composition, the macroscopic response of metamaterials is dictated by their sophisticatedly engineered architectures [3]. Over the past decade, research in this field has transitioned from theoretical exploration to practical implementation, enabling unprecedented mechanical signatures including negative Poisson's ratios [4], and bistable switching [5]. These breakthroughs have catalyzed transformative applications across diverse sectors, ranging from energy‐absorbing impact protectors [6, 7, 8, 9], soft robotics [10, 11, 12, 13, 14], and tissue engineering scaffolds [15, 16, 17, 18, 19]. As the demand for multi‐functional and extreme‐performance materials intensifies, a key research topic has focused on identifying architectural paradigms that can effectively integrate high load‐bearing capacity with high energy absorption and structural isotropy [20, 21].

To address these needs, researchers have increasingly turned to nature as a premier source of inspiration [22, 23]. Evolution‐refined biological structures offer a vast library of high‐performance blueprints that have been optimized over millions of years to withstand complex environmental loading [23]. By translating these natural prototypes into mechanical metamaterials, engineers have successfully developed architectures that surpass conventional solids in fracture toughness [6, 24, 25], stiffness tunability [10, 26], and programmable deformation [27, 28]. From the hierarchical nacre‐like structures to the lightweight lattice‐like trabecular bone, bio‐inspired design has provided a robust route to achieving superior mechanical efficiency that synthetic periodic lattices often struggle to match [22, 23].

Yet, a critical gap persists between biological reality and engineering abstraction. Most bio‐inspired designs have historically focused on pristine periodic lattices, simplifying the intrinsic complexity of nature for the sake of computational and manufacturing convenience [25, 29, 30, 31]. However, many outstanding biological materials—such as bone, wood, and cuttlebone—are inherently disordered at micro‐ to meso‐scales [32, 33, 34]. This randomness is not a defect but a functional feature; recent studies indicate that disordered metamaterials can exhibit unique advantages, including defect insensitivity [35, 36, 37], enhanced damage tolerance [38, 39, 40], and unconventional stiffness‐density scaling laws [41], thereby holding significant potential in robustness and energy absorption.

Existing strategies for engineering disordered metamaterials primarily focus on stochastic node perturbation and element removal in periodic structures [36, 40, 42, 43, 44, 45], and virtual growth algorithms [35, 46], which typically yield continuous network structures. This leaves an entire architectural domain—discrete disorder—largely unexplored. Nature, by contrast, exploits discrete disorder in structures like the cuttlebone, a fragmented assemblage of chambered walls that delivers an exceptional triad of specific strength, stiffness, and resilience [47]. To date, biomimetic studies have largely periodized this geometry [28, 48, 49, 50, 51, 52], stripping away the randomness whose fundamental mechanical purpose remains a significant question in structural biology.

Herein, we propose a biomimetic cuttlebone structures (named as BCS) whose disorder is dialed by a single parameter *α*. 3‐D‐printed samples spanning *α* = 0–1 were tested in compression and shear, and modelled at high resolution. Rising *α* triggers localized buckling, multiplies internal contacts and randomizes orientations, simultaneously boosting buckling resistance, energy absorption and shear isotropy. Benchmarked against state‐of‐the‐art lattices, fully disordered BCS (*α* = 1) delivers the specific strength/*E*_s_ and specific energy absorption (SEA)/*E*_s_, offering a direct route to lighter, tougher and more efficient energy‐absorbing structures.

## 2 Design Principles

### 2.1 Architecture and Mechanics of Natural Cuttlebone

**FIGURE 1:**
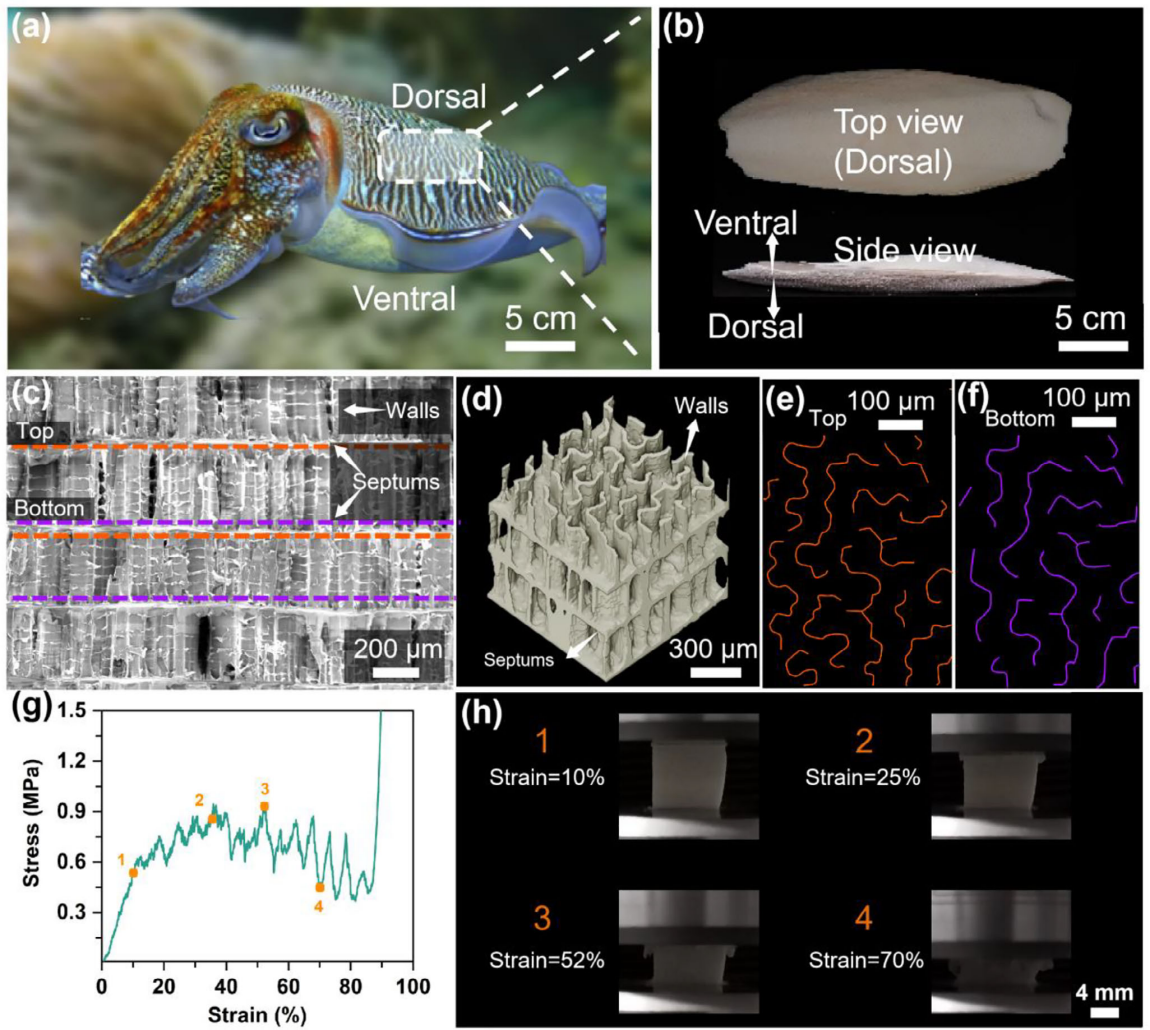
**Architecture and mechanics of natural cuttlebone**. (a) Cuttlefish (*Sepia officinalis*). (b) Excised cuttlebone, dorsal view. (c) SEM micrograph showing horizontal septums and discrete vertical walls. (d) Micro‐CT volume rendering; chambers and struts resolved isotropically. (e) Top and (f) bottom contours of a representative vertical wall, revealing gradient amplitude. (g) Quasi‐static uniaxial stress–strain response; linear elasticity, plateau and densification regimes labeled. (h) Recorded sequence of progressive layer‐by‐layer collapse during compression. (view normal to loading axis).

The high‐pressure environment of the deep sea raises unique challenges for biological materials. The cuttlebone, located along the dorsal side of cuttlefish (Figure 1a,b), represents an outcome of adaptive evolution. Despite its very low mass and relative density, the cuttlebone sustains hydrostatic loads that would implode engineered lattices of equal density. X‐ray micro‐computed tomography (micro‐CT) and scanning electron microscopy (SEM) expose its architecture (Figure 1c,d): horizontal septums seal off an array of gas‐filled chambers, each partitioned by discrete, vertically oriented walls. These walls are characterized primarily by a wavy morphology with a form close to sinusoidal, combined with a non‐periodic arrangement; Their wave amplitudes grow continuously from bottom to top (Figure 1e,f), and lengths scatter in a distribution that appears random. Cubic samples measuring 1 × 1 × 1 cm were excised by a wire‐cut electrical discharge machine (EDM) compressed in three distinct regimes—elastic, plateau, densification—yet the brittle calcite skeleton (Figure 1g) yielded like a ductile foam. Serrated plateaus manifested layer‐by‐layer wall collapse (Figure 1h), while lateral strain remained essentially unchanged, demonstrating extraordinary stability. These observations indicate that the internal disordered architecture of cuttlebone plays a decisive role in determining its mechanical performance. Although idealized periodic models can simplify structural design, neglecting inherent structural disorder risks overlooking the key mechanical advantages and underlying mechanisms conferred by such randomness.

### 2.2 Biomimetic Cuttlebone Structure (BCS) Design

**FIGURE 2:**
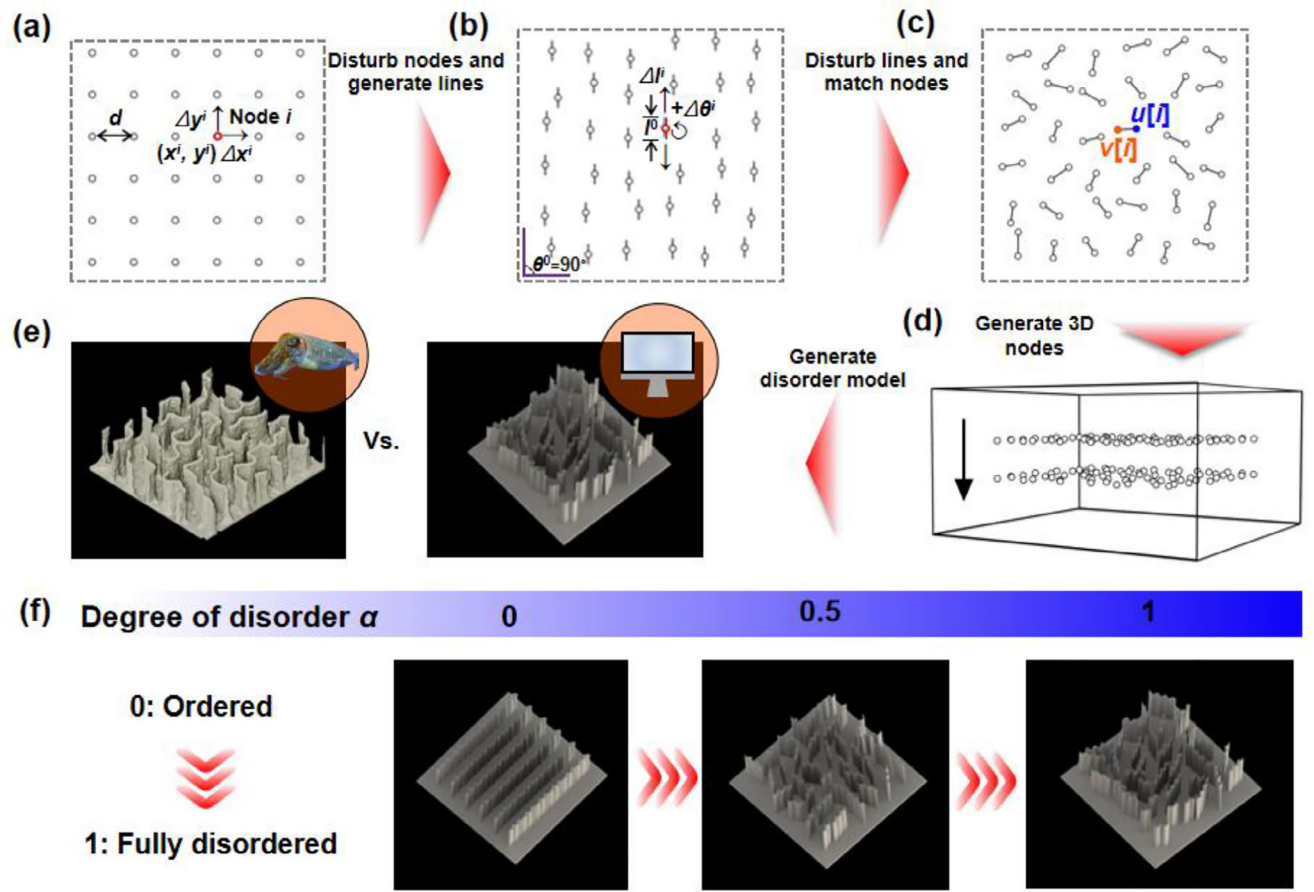
**Generation procedure of the biomimetic cuttlebone structure (BCS)**. (a) Regular nodal grid and stochastically perturbed node positions: each node *i* is displaced by random positional perturbations (*Δx^i^*, *Δy^i^*) from its initial coordinates (*x^i^*, *y^i^*). (b) Line segments grown from each perturbed node *i*, all with a common initial vertical angle *θ* ^0^ = 90°, and each segment *i* subjected to random length perturbation *Δl^i^* and random angular perturbation *Δθ^i^* (rotated about node *i*) to introduce directional disorder. (c) End nodes *u*[*i*] and *v*[*i*] created and indexed for each segment. (d) Indexed nodes extruded vertically to height *h* to construct the 3‐D BCS lattice. (e) Natural cuttlebone (left) and rendered BCS model (right) showing matched waviness and discrete walls. (f) BCS realizations for *α* = 0, 0.5, 1, illustrating the continuum from periodic to fully disordered architectures.

To systematically dissect how disorder governs the mechanics of cuttlebone‐like architectures, we devised a generator that tunes randomness with single‐parameter precision (Figure 2). Beginning with a 6 × 6 grid of nodes spaced at distance *d* (Figure 2a), we introduced the scalar *α* ∈ [0, 1]—adopted from Romijn & Fleck [53]—to calibrate the level of disorder. Each node *i* with coordinates (*x^i^*, *y^i^*) was offset by (*Δx^i^*, *Δy^i^*), as prescribed by topological constraint Equations (1), (2). This process generated a spectrum of BCS geometries that interpolated between crystalline order and stochastic fragmentation while maintaining connectivity.

(1) $$\Delta x^{i}={\bar{x}}^{i}-x^{i}=\beta \alpha r$$

(2) $$\Delta y^{i}={\bar{y}}^{i}-y^{i}=\beta \alpha r$$

where (${\bar{x}}^{i}$, ${\bar{y}}^{i}$) represents the coordinates of the perturbed nodes, respectively. *β*(− 1 ≤ *β* ≤ +1) represents random variable, and *r* represents the maximum allowable perturbation value, given by *r* = 0.25*d*. Using the displaced nodes *i* as centers, line segments of a common initial length *l* ^0^ were generated. All segments shared a common initial vertical angle *θ* ^0^ = 90°, with counterclockwise rotation about node *i* defined as positive. Subsequently, the angles of these segments were perturbed by *Δθ^i^* based on topological constraint Equation (3) (Figure 2b) to introduce directional disorder mimicking that of the cuttlebone.

(3) $$\Delta \theta^{i}={\bar{\theta }}^{i}-\theta^{0}=180\beta \alpha$$

where ${\bar{\theta }}^{i}$ denotes the perturbed angle of segment *i*. Following this, the segment lengths were perturbed *Δl^i^* according to topological constraint Equation (4), with nodes numbered *v*[*i*] and *u*[*i*] created at both ends of each segment (Figure 2c):

(4) $$\Delta l^{i}={\bar{l}}^{i}-l^{0}=\beta \alpha s$$

where ${\bar{l}}^{i}$ denotes the perturbed length of segment *i*, and *s* represents the maximum allowable length perturbation, given by *s* = 0.5*l* ^0^.

Corresponding nodes were then extruded longitudinally at height *h* to create the 3‐D scaffold (Figure 2d). Each vertical strut was shaped as a sinusoid whose amplitude grows linearly from bottom to top (Figure S1). All struts shared a common initial top amplitude $A_{\mathrm{top}}^{0}$. To mirror the natural scatter seen in cuttlebone, amplitude disorder was imposed via:

(5) $$\Delta A_{\mathrm{top}}^{i}={\bar{A}}_{\mathrm{top}}^{i}-A_{\mathrm{top}}^{0}=\beta \alpha p$$

where ${\bar{A}}_{\mathrm{top}}^{i}$ denotes the perturbed top amplitude of strut *i*, and *p* represents the maximum allowable amplitude perturbation, given by *p* = 0.5$A_{\mathrm{top}}^{0}$.

The range for the ratio of top‐to‐bottom amplitude was based on observations from the real cuttlebone (Table S1). A baseline ratio of 3.5 was selected because within the observed range, the compressive strength, Young's modulus, and SEA of the units increased monotonically with a higher top‐to‐bottom amplitude ratio (Figure S2). Choosing this intermediate value helped to minimize the influence of amplitude variations on mechanical performance.

A uniform wall thickness *t*_w_ was finally assigned to the generated profile, completing the disordered BCS (Figure 2e); geometric details are listed in Table S2. All wall units remain fully discrete and unconnected for all *α*, ensuring consistent connectivity, effective wall length, and fair comparison between ordered and disordered structures. Side‐by‐side comparison with the natural cuttlebone confirmed close morphological fidelity. Sweeping *α* from 0 to 1 drove the architecture from perfectly periodic to fully stochastic (Figure 2f), with statistical distributions of all structural metrics flattening and broadening as disorder grows (Figure S3). Despite this geometric spread, the relative densities of the three extremes differed by < 0.4% (13.3%, 13.7%, 13.7%), verifying that *α* governs geometry, not density.

Unlike the unique *α =* 0 lattice, any *α* > 0 yields an ensemble of distinct geometric configurations. To ensure statistical robustness and experimental reproducibility, we simulated quasi‐static compression on 64 independent structural realizations of fully disordered BCS (*α* = 1) with 4, 16, and 36 unit cells (Figure S4). Stress‐strain traces converged as the unit count rose (Figure S4a–c), and the coefficient of variation for strength, modulus and energy absorption dropped below 10% once 36 units are used (Figure S4d). This 6 × 6 array was therefore adopted as the minimal representative volume element for all subsequent tests.

## 3 Results and Discussion

### 3.1 Compressive Behavior

**FIGURE 3:**
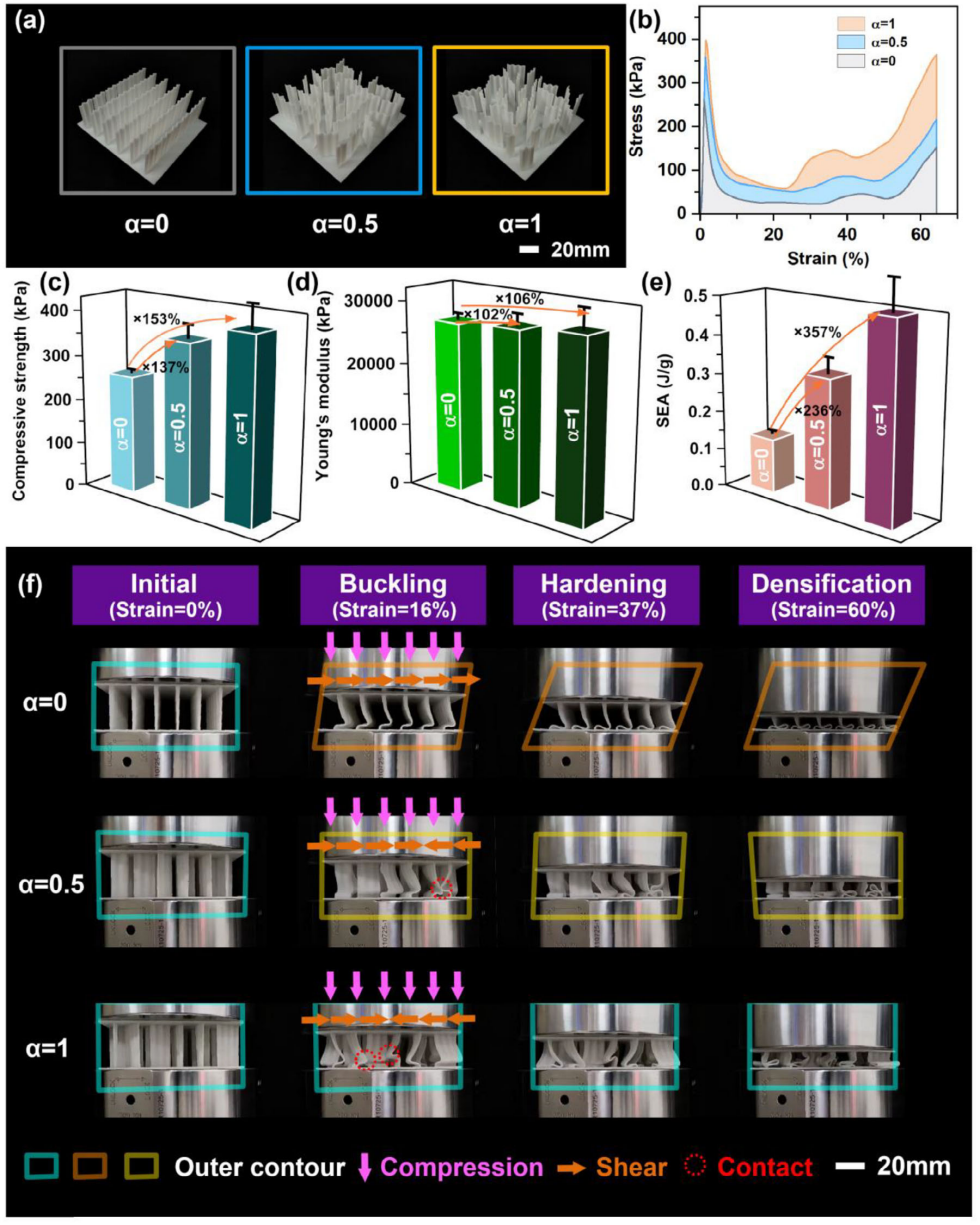
**Quasi‐static compression of 3‐D‐printed BCS**. (a) As‐printed lattices with *α* = 0, 0.5, 1. (b) Nominal stress–strain responses; disorder elevates peak and plateau stresses. (c) Compressive strength, (d) Young's modulus and (e) specific energy absorption versus *α*, (f) High‐speed snapshots of the deformation sequence; global shear bands at *α* = 0 evolve into stable, layer‐wise collapse at *α* = 1.

36‐unit BCS replicas with *α* = 0, 0.5, and 1 were additively manufactured from photopolymer resin (Figure 3a) and tested under quasi‐static compression (definitions of all metrics are given in the Supporting Information). Every curve (Figure 3b) traced the canonical sequence—linear elasticity, buckling‐induced stress drop, extended plateau, late‐stage hardening and final densification—mirroring the ductile response of the parent resin. Peak and plateau stresses, however, scaled directly with disorder: the fully disordered lattice delivered a plateau stress 2.95× that of its periodic counterpart (Figure S5a). Crush force efficiency (*λ*) and smoothness coefficient (*η*) rose similarly, by factors of 1.88 and 2.12 respectively (Figure S5b, c), confirming that randomness suppresses catastrophic collapse and stabilizes energy absorption throughout the plateau.

Compressive strength, Young's modulus, and SEA all scale with *α* (Figure 3c–e). At *α* = 1 the gains were + 53%, + 6% and + 257%, respectively, relative to the periodic lattice. The modest rise in modulus reflects the combined influence of geometric diversity and minor deviations in relative density—factors that can mask the intrinsic effect of disorder. To isolate them we decomposed each 36‐unit lattice into its constituent cells, linearly superposed their individual force–displacement responses and recomputed the global properties (Figure S6a–c). After this decoupling, strength and SEA still outpaced the periodic reference by ≈ 41% and 206%, whereas the modulus remained unchanged. (Figure S6d). Thus disorder retards buckling and amplifies energy absorption. Remarkably, this buckling‐toughening effect is the opposite of that seen in continuous network metamaterials, where randomness typically seeds premature failure; discrete walls appear to redistribute, rather than concentrate, destabilizing loads.

Figure 3f illustrates the deformation modes of BCS during compression. In the periodic BCS (*α* = 0), internal unit cells collapsed in a coordinated directional manner, leading to global synchronous buckling. Such uniform buckling mode was gradually suppressed with increasing disorder. The fully disordered BCS (*α* = 1) maintained a stable deformation pattern throughout compression, consistent with the behavior observed in natural cuttlebone samples. This stabilization arose from the transition from periodic to disordered architecture, which diversified structural features and disrupted the coordinated buckling of unit cells, allowing shear components from different directions to mutually cancel. Simultaneously, Out‐of‐phase buckling between adjacent walls induced contact between opposing surfaces; these self‐generated contacts enhanced the resistance to further compression. The friction arising at the contact interfaces further improved energy dissipation efficiency, while continued compression drove the buckled segments against the horizontal septums, with stress gradually intensifying until the structure achieved full densification.

**FIGURE 4:**
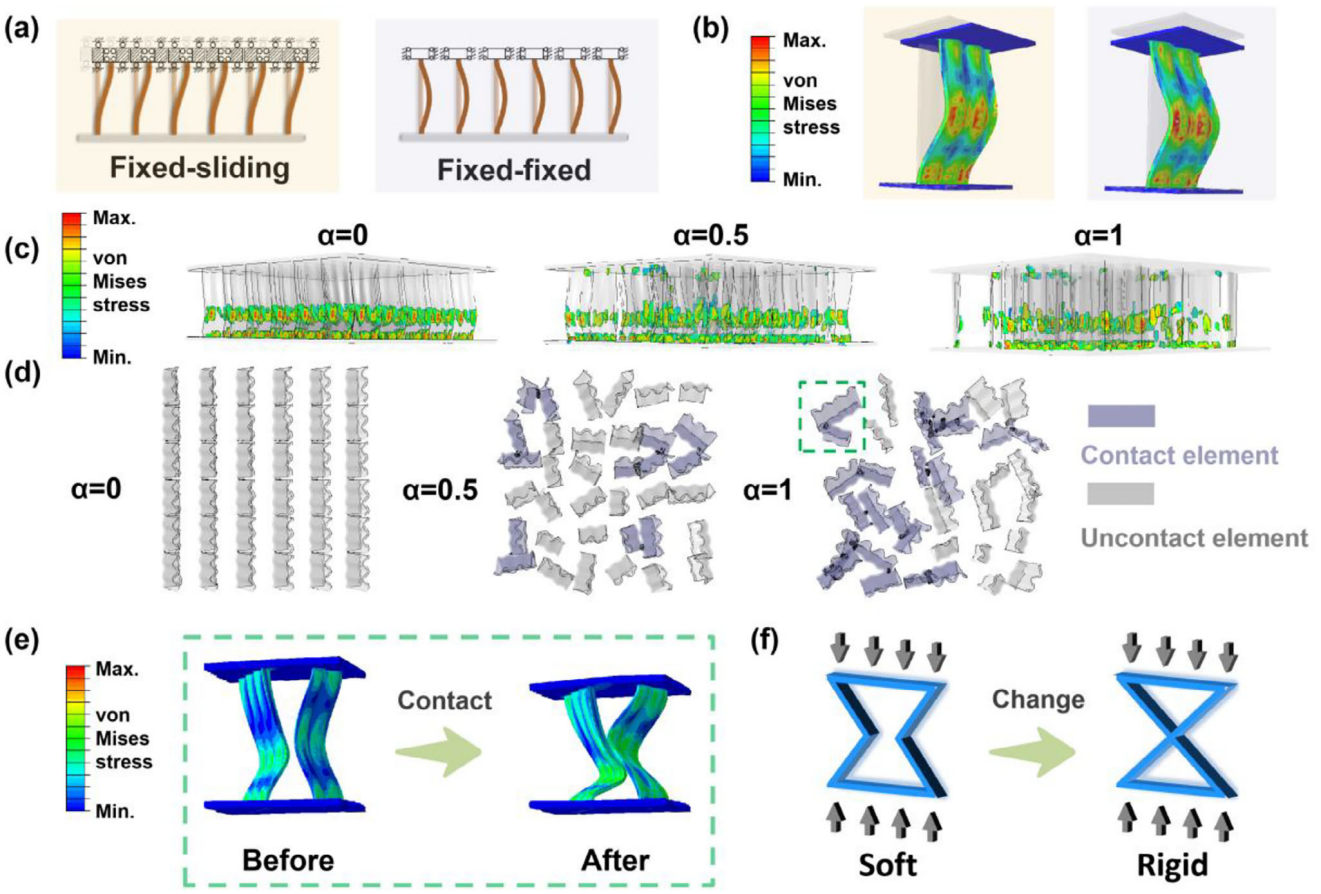
**Finite‐element analysis of BCS**. (a) Equivalent boundary constraint models for periodic (fixed‐sliding) and disordered (fixed‐fixed) unit cells. (b) Distinct buckling behaviors and stress distributions under fixed‐sliding vs. fixed‐fixed constraints. (c) Full‐lattice von Mises stress fields showing mixed buckling modes with increasing *α*. (d) Self‐contact formation during the plateau stage, with contact element count scaling with *α*. (e) Stress redistribution in contacting unit pairs before and after self‐contact. (f) Schematic of the flexible‐to‐rigid transition driven by self‐contact.

Finite‐element snapshots expose why randomness hardens the lattice (Figure 4). Building on the experimental observations of compressive collapse, the upper and lower boundaries of periodic (*α* = 0) and disordered (*α* = 1) BCS can be simplified to fixed‐sliding and fixed‐fixed constraints [54], respectively (Figure 4a). Simulations of identical unit cells under these two distinct boundary conditions revealed markedly different buckling behaviors: relative to the fixed‐sliding constraint, where stress was confined to the base and mid‐height, the fixed‐fixed constraint introduces an additional high‐stress region at the top surface (Figure 4b). Correspondingly, the fixed‐fixed unit cell stored significantly higher elastic energy than its fixed‐sliding counterpart (Figure S7), which explains why disordered structures exhibit superior strength compared to periodic ones. Across the full 6 × 6 array, individual unit cells adopted a mixed mode of these two buckling patterns. As *α* increased, the proportion of unit cells exhibiting the fixed‐fixed buckling mode rose progressively (Figure 4c), which verified that enhanced disorder strengthened the lateral constraint between adjacent unit cells. With further compression, self‐contact emerged between neighboring walls (Figure 4d). In the periodic BCS, no contact occurred between internal unit cells; in contrast, self‐contact initiated early in the plateau stage for disordered configurations, and the number of contacting unit pairs increased directly with *α*. A examination of the stress distribution in contacting unit pairs demonstrated that self‐contact induced effective stress redistribution (Figure 4e), resulting in a more uniform stress field across the structure. This improvement arose because the contacting unit pairs underwent a transition from a flexible framework to a rigid one (Figure 4f) [55, 56], with the deformation mechanism shifting from bending‐dominated to compression‐dominated loading—an evolution that underpinned the high plateau stress of disordered BCS.

### 3.2 Shear Response

**FIGURE 5:**
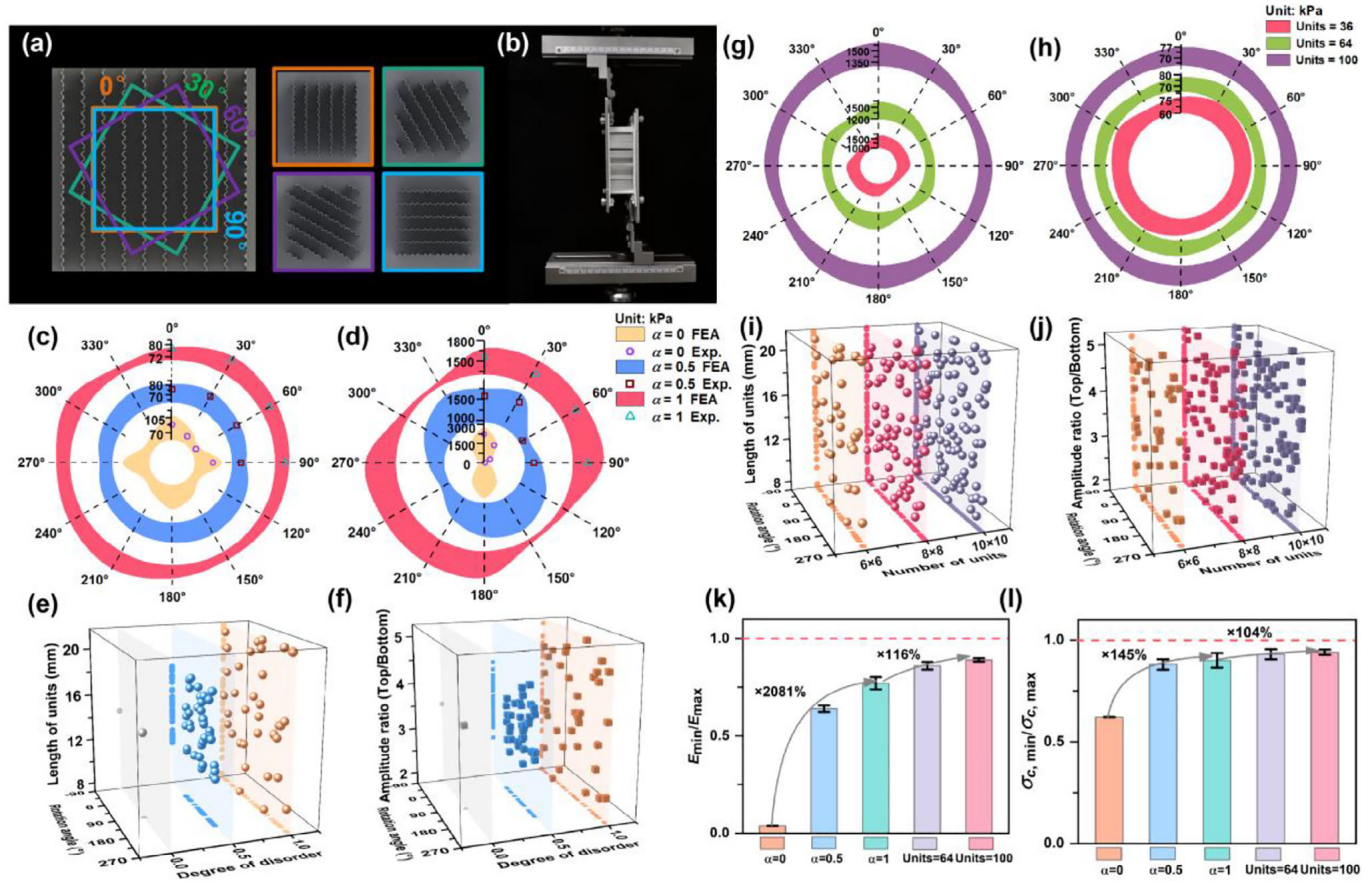
**Shear response of BCS**. (a) Concept: 6 × 6 domain rotated in 30° increments for directional shear. (b) Experimental setup for the shear. (c) Polar plot of shear strength and (d) shear modulus versus loading angle for *α* = 0, 0.5, 1. (e) Scatter plot of unit length and (f) amplitude ratio across all directions; distributions evolve toward full spatial uniformity as *α* increases. (g) Shear modulus and (h) strength for fully disordered lattices with 6 × 6, 8 × 8 and 10 × 10 unit counts. (i, j) Corresponding rose plots showing further homogenization of length and amplitude with larger unit counts. (k) *E*_min_/*E*_max_ and (l) *σ*_c, min_/*σ*_c, max_ versus disorder and unit count; dashed line marks ideal isotropy (value = 1).

In the abyss, cuttlefish endure omnidirectional shear from shifting currents and self‐propelled manoeuvres; their skeleton must therefore offer near‐isotropic stability. To quantify this capability, a 6 × 6 unit domain was rotated in 30° increments (0–360°) and analyzed under simple shear (Figure 5a). Identical orientations were machined from 3D printed blocks and tested in a custom fixture (Figure 5b). Periodic BCS (*α* = 0) displayed pronounced directional sensitivity: shear strength and modulus varied by ± 23% and ± 93%, respectively, with orientation (Figure 5c,d). Increasing *α* systematically flattened both polar diagrams; at *α* = 1 the variation had fallen to ± 5% in strength and ± 13% in modulus, demonstrating that disorder converts the lattice into an effectively isotropic shear medium.

Anisotropic is rooted in directional bias of two micro‐metrics: wall length and waviness amplitude. In the periodic solid these metrics are identical in every cell, so any rotation of the shear plane immediately samples a new, un‐effective projection—hence the sharp angular variation. Disorder removes the bias: distribution of unit length and amplitude evolved toward full spatial uniformity as *α* → 1 (Figure 5e,f), endowing each direction with statistically equivalent load‐bearing area. Enlarging the sample amplified this averaging effect. Fully disordered 6 × 6, 8 × 8 and 10 × 10 domains were sheared at 30° intervals; strength and modulus scatter dropped from ± 5% and ± 13% to ± 3% ± 6% as the count rose (Figure 5g,h). Polar histograms confirm that 10 × 10 lattices approached perfect isotropy because the additional cells further smoothed the already narrowed distributions of length and amplitude (Figure 5i,j).

Shear anisotropy is quantified by the directional min‐to‐max ratios *E*_min_/*E*_max_ and *σ*_c, min_/*σ*_c, max_ (a value of 1 denotes perfect isotropy) [21]. Varying only *α*, the fully disordered BCS gave an *E*_min_/*E*_max_ 20.81× higher than its periodic counterpart, whereas *σ*_c,min_/*σ*_c,max_ already exceeded 0.9 (Figure 5k,l). Expanding the lattice from 6 × 6 to 10 × 10 pushed these ratios an additional 16% and 4% closer to unity, confirming that both higher disorder and larger sample size drive the architecture toward ideal shear isotropy.

### 3.3 Multi‐Layer BCS

**FIGURE 6:**
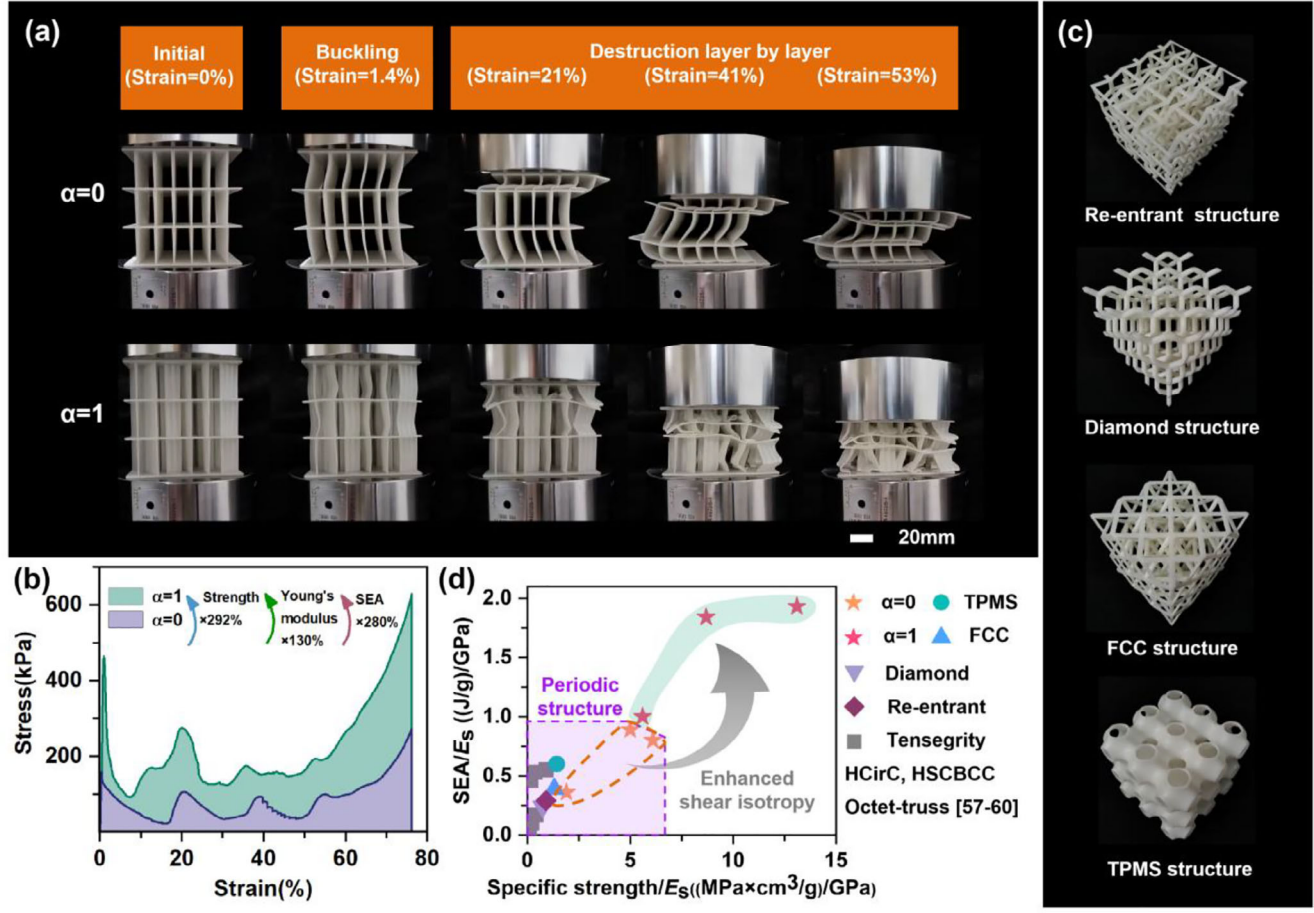
**Multi‐layer BCS under quasi‐static compression**. (a) High‐speed sequences showing layer‐by‐layer collapse; periodic stack (left) kinks globally, disordered stack (right) remains stable. (b) Corresponding stress–strain curves; serration count equals layer number and disorder elevates peak and plateau stresses. (c) As‐printed re‐entrant, diamond, FCC and TPMS lattices produced with identical resin and process parameters. (d) Ashby plot of specific strength/*E*_s_ and SEA/*E*_s_; fully disordered BCS (red star) outperforms all benchmark periodic architectures and delivers near‐isotropic shear.

Stacking three single‐layer slabs yielded 3‐periodic and 3‐disordered lattices that mimicked the natural cuttlebone's tiered architecture. Both variants collapsed layer‐by‐layer, a failure mode similar to that of natural cuttlebone, producing stress–strain serrations whose number equals the layer count (Figure 6a,b and Movie S1). The disordered stack, however, remained laterally stable (Movie S1), whereas the periodic copy developed catastrophic one‐sided kinking (Movie S2). Strength, modulus and SEA of the fully disordered stack exceeded those of its periodic counterpart by 2.92×, 1.30× and 2.80×—gains larger than in the single‐layer case. Height amplifies imperfection sensitivity; repeating units provide a ready path for buckle propagation, while the stochastic interior of the disordered lattice scatters loads in multiple directions and arrests global tilt. Maintaining constant relative density, we next varied the wall‐to‐septum thickness ratio *t*_w_/*t*_s_ = 0.5, 1, 2 (Figure S8). Raising *t*_w_/*t*_s_ monotonically increased strength and modulus for both architectures, but SEA plateaued: a jump from 1 → 2 added 9% to the disordered lattice yet lowered the periodic one by 24%. A judicious choice of *t*_w_/*t*_s_ therefore lets the disordered BCS trade modest weight for disproportionate gains in strength and energy absorption.

An Ashby map positions the BCS against eight benchmark lattices—TPMS, FCC, diamond, re‐entrant, HCirC [57], Tensegrity [58], HSCBCC [59], and Octet‐truss (Figure 6d) [60]. TPMS, FCC, diamond, and re‐entrant structures were fabricated using the same process as the designed BCS (Figure 6c); all data are normalized by the solid Young's modulus (*E*_s_) to remove material bias [48, 61, 62]. Fully disordered BCS (*α* = 1) occupied the upper‐right quadrant, simultaneously delivering the highest specific strength/*E*_s_ and SEA/*E*_s_ of the cohort while offering near‐isotropic shear response. The plot confirms that topological randomness lifts the cuttlebone motif into a region inaccessible to current periodic metamaterials.

## 4 Conclusions

By unravelling how stochastic architecture governs the mechanics of discrete solids, we have translated the cuttlebone's disordered structure into a tunable metamaterial. A single parameter *α* dials the lattice from perfect periodicity to full randomness while holding relative density constant; at *α* = 1 the resulting biomimetic cuttlebone structure delivers 41% higher specific strength and 206% greater specific energy absorption than its periodic counterpart, doubles the plateau stress, and suppresses global buckling. Disorder also erases shear anisotropy—the ratio of minimum to maximum shear stiffness (*E*_min_/*E*_max_) rises by 1981%, while the shear‐strength ratio (*σ*_c, min_/*σ*_c, max_) exceeds 0.9—yielding an isotropic, damage‐tolerant solid that collapses layer‐by‐layer exactly like its natural template. These findings establish discrete randomness as a previously under‐exploited design axis and open a direct route to lighter, safer protective systems for aerospace, automotive and marine applications.

## 5 Experimental Section

### 5.1 Specimen Fabrication

Cuttlefish obtained from Zhangzhou, China, and dissected to harvest intact cuttlebones. After removal, the cuttlebones were allowed to dry naturally in the laboratory at 25°C for 72 h until constant mass was achieved. Cubic specimens (10 × 10 × 10 mm^3^) were then sectioned with a diamond wire saw, orienting the septums perpendicular to the subsequent compression axis.

For lattices with *α* of 0.5 and 1, five distinct geometric configurations were designed per *α*, and three replicate samples were fabricated for each configuration, yielding 15 samples per disorder level. For the periodic lattice (*α* = 0), only three replicates were prepared, as all periodic structures were geometrically identical.

Benchmark lattices (TPMS, FCC, re‐entrant, and diamond structures) were drafted in CAD and built on a Stereolithography Apparatus (SLA) 3D printer (UnionTech RS6000, UnionTech Co., Ltd., China) with a commercial photosensitive resin (Somos EvoLVe 128, DSM, Netherlands). Three replicates were fabricated for each benchmark lattice using the same processing parameters. The layer thickness, contour speed, and fill speed were 0.1 mm, 4000 mm/s, and 8000 mm/s, respectively. After printing, parts were ultrasonically rinsed in isopropanol, UV‐post‐cured for 2 h to complete cross‐linking, depinned and edge‐polished to eliminate stress concentrators.

### 5.2 Sample Characterization

Thin slices were dry‐cut from the conditioned cuttlebone, mounted on a aluminum stubs, and sputter‐coated with ≈ 10 nm Au. Microstructure was imaged in a SEM (Zeiss Gemini 360, Carl Zeiss, Germany) at an accelerating voltage of 5 kV.

The cubic cuttlebone sample was scanned non‐destructively in an micro‐CT (Zeiss Xradia 520, Carl Zeiss, Germany) at 50.12 kV, 158.36 µA, 5.2 µm voxel size and 1 s exposure. Tomograms were reconstructed in Avizo software (Version 2019.1, Thermo Fisher Scientific, USA); The key morphological metrics (wall thickness and amplitude) were listed in Table S1.

### 5.3 Mechanical Tests

Uniaxial quasi‐static compression tests were conducted at room temperature and a strain rate of 10^−^^3^/s on an Instron 68SC‐1 (cuttlebone, Instron, USA) or Instron 5985 (printed lattices, Instron, USA), with the loading axis perpendicular to the cuttlebone septums. Load, displacement and synchronized images were recorded to construct nominal stress–strain curves.

Shear tests used a custom‐built tensile‐shear fixture (Figure S9, see Supporting Information for details) mounted in an Instron 5985 (Instron, USA). Samples were centered on the fixture axis to eliminate bending, then sheared at 10^−^^3^ s^−^^1^ while shear stress–strain data were logged.

### 5.4 Finite‐Element Simulation

Finite‐element simulations were performed in Abaqus/Explicit (Version 2020, Dassault Systèmes, France) to capture large deformation, self‐contact and material non‐linearity under quasi‐static conditions. Kinetic energy was monitored and kept below 5% of internal energy to ensure a quasi‐static response. An elastic–plastic constitutive model was assigned (*E* = 700 MPa, *σ*_y_ = 14.5 MPa, *ρ* = 1.12 g cm^−3^, *ν* = 0.3). The lattice was discretized with 10‐node quadratic tetrahedra (C3D10M) to mitigate shear locking and accommodate severe mesh distortion. Predictions of compressive strength, Young's modulus and SEA agreed within 15% of experimental values (Figure S10), validating the model for parametric and mechanistic studies. For both compression and shear simulations, five distinct geometric configurations were selected for each disorder level (*α =* 0.5 and 1), corresponding to the representative configurations employed in experiments.

## Author Contributions

**Zengqin Shi**: Investigation, methodology, writing – original draft. **Peng Jiang**: Investigation, data curation. **Xiaoyan Zhu**: Conceptualization, formal analysis. **Suyun Li**: Visualization, validation. **Wenqing Wang**: Visualization, investigation. **Tian Zhao**: Visualization, investigation, supervision. **Rujie He**: Writing – review & editing, supervision, funding acquisition. **Ying Li**: Supervision, funding acquisition.

## Conflicts of Interest

The authors declare no conflicts of interest.

## Supporting information




**Supporting File 1**: advs75605‐sup‐0001‐SuppMat.docx.

**Supporting File 2**: advs75605‐sup‐0002‐MovieS1.mp4.

**Supporting File 3**: advs75605‐sup‐0003‐MovieS2.mp4.

## Data Availability

The data that support the findings of this study are available from the corresponding author upon reasonable request.
