# Supplementary material for: Unraveling the Power of Disorder: A Bioinspired Cuttlebone Structure With Superior Strength, Energy Absorption and Isotropy
Source: Adv Sci (Weinh). 2026 May 8;13(43):e75605. doi: 10.1002/advs.75605 (PMC13335895; doi:10.1002/advs.75605)
Supplement: Supplementary file 1 — Supporting File 1: advs75605‐sup‐0001‐SuppMat.docx. [file ADVS-13-e75605-s003.docx]

Supporting Information

**Unraveling the Power of Disorder: A Bioinspired Cuttlebone Structure with Superior Strength, Energy Absorption and** **Isotropy**

*Zengqin Shi^1^, Peng Jiang^1^, Xiaoyan Zhu^3, 4^, Suyun Li^1^, Wenqing Wang^1^, Tian Zhao^1^, Rujie He^1, 2^*, Ying Li^1, 2^**

*^1^ Institute of Advanced Structure Technology, Beijing Institute of Technology, Beijing 100081, China*

*^2^ Marine Science and Technology Domain, Beijing Institute of Technology, Zhuhai 519088, China*

*^3^ School of Continuing and Lifelong Education, National University of Singapore, Singapore 119077, Singapore*

*^4^ Institute of Systems Science, National University of Singapore, Singapore 119615, Singapore*

*__________*

****Corresponding authors***

*Rujie He, herujie@bit.edu.cn*

*Ying Li, bitliying@bit.edu.cn*

**Mechanical metrics**

Specific energy absorption (SEA), a critical index for crash-worthiness and blast mitigation, was computed from the stress–strain curves as the energy absorbed per unit mass up to densification:

$\text{SEA}=\frac{\int_{0}^{\varepsilon_{\text{d}}} \sigma(\varepsilon)\text{d}\varepsilon}{\rho^{*}}$ (1)

$\rho^{*}=\rho_{\text{r}}\rho_{\text{s}}$ (2)

where *σ* and *ε* are nominal stress and strain. *ε*_d_ (= 0.5 for all samples to ensure consistent comparison) is the densification strain. *ρ^*^*, *ρ*_r_, and *ρ*_s_ denote the effective, density, and solid densities, respectively.

Plateau stress (*σ*_m_) is the average engineering stress maintained during progressive collapse. It quantifies the sustained load-bearing capacity while the lattice undergoes plastic buckling, fracture or densification, and is calculated as:

$\sigma_{\text{m}}=\frac{\int_{\varepsilon_{i}}^{\varepsilon_{\text{d}}} \sigma(\varepsilon)\text{d}\varepsilon}{\varepsilon_{\text{d}}-\varepsilon_{i}}$ (3)

where *ε_i_* represents the initial strain of the plateau stage.

Crush-force efficiency (*λ*) reflects the moderation of stress decline after the elastic stage, defined as the ratio of the plateau stress to the initial peak stress (*σ*_m_*/σ*_p_). A higher value indicates that the material maintains a higher load-bearing capacity after yielding, avoiding a sharp stress drop. It can be expressed as:

$\lambda=\frac{\sigma_{\text{m}}}{\sigma_{\text{p}}}$ (4)

*σ*_p_ denotes the initial peak stress.

Smoothness coefficient, *η*, quantifies the flatness of the post-yield stress–strain path; higher *η* denotes a steadier plateau with fewer stress serrations and more uniform energy absorption:

$\eta=\frac{\sigma_{\text{m}}}{\sigma_{\max}-\sigma_{\min}}$ (5)

where *σ*_max_ and *σ*_min_ are the maximum and minimum stresses recorded during the post-yield stage, respectively.

Table S1. Structural parameters of the Cuttlebone.

| Structural parameters (Cuttlebone) | Value (μm) |
| --- | --- |
| Walls thickness | 5-9 |
| Septums thickness | 10-17 |
| Bottom amplitude | 8-26 |
| Top amplitude | 14-42 |

Table S2. Structural parameters of the BCS.

| Structural parameters (BCS) | Value (mm) |
| --- | --- |
| Initial nodes spacing *d* | 16 |
| Initial length *l*^0^ | 14.4 |
| Height *h* | 32 |
| Thickness *t*_w_ | 0.8 |
| Bottom amplitude *A*_bottom_ | 0.32 |


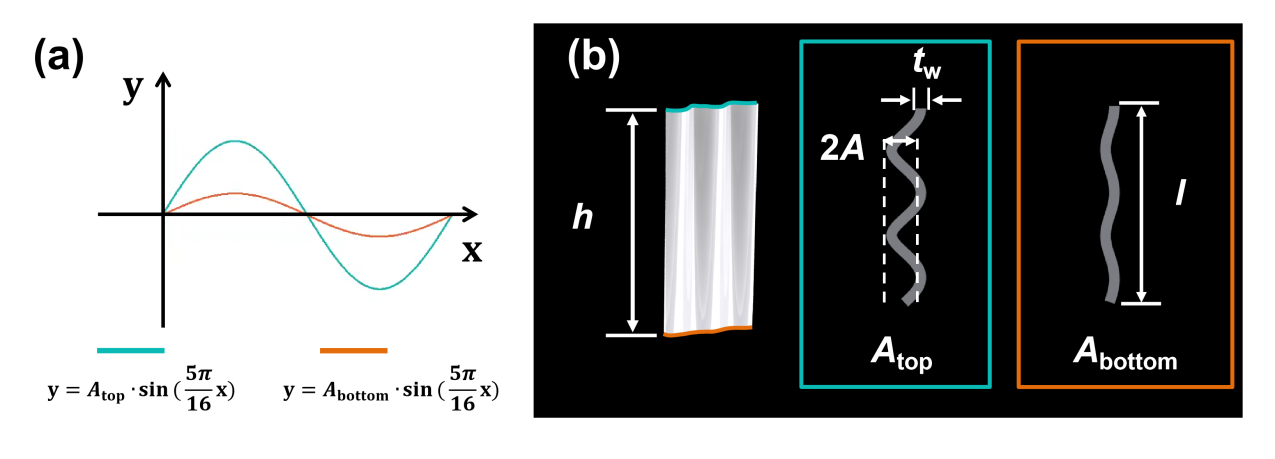


**Figure** **S1. BCS geometric definition.** (a) Sinusoidal profile at bottom (amplitude *A*_bottom_) and top (amplitude *A*_top_). (b) Key unit-level parameters: wavelength *A*, wall length *l*, thickness *t*_w_ and mapping height *h*.


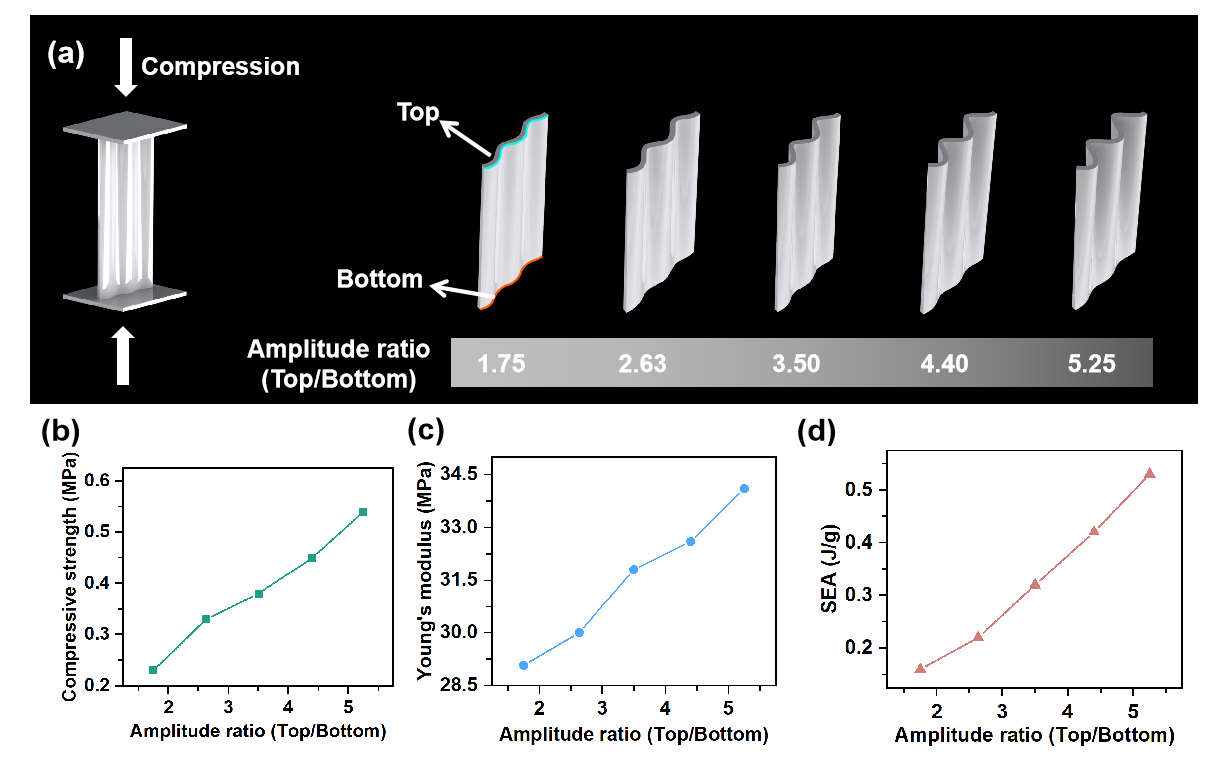


**Figure S2. Influence of amplitude gradient on single-unit mechanics.** (a) Unit cells with top-to-bottom amplitude ratios of 1.75, 2.63, 3.50, 4.40 and 5.25. (b) Compressive strength, (c) Young’s modulus and (d) SEA versus amplitude ratio; all metrics increase monotonically.


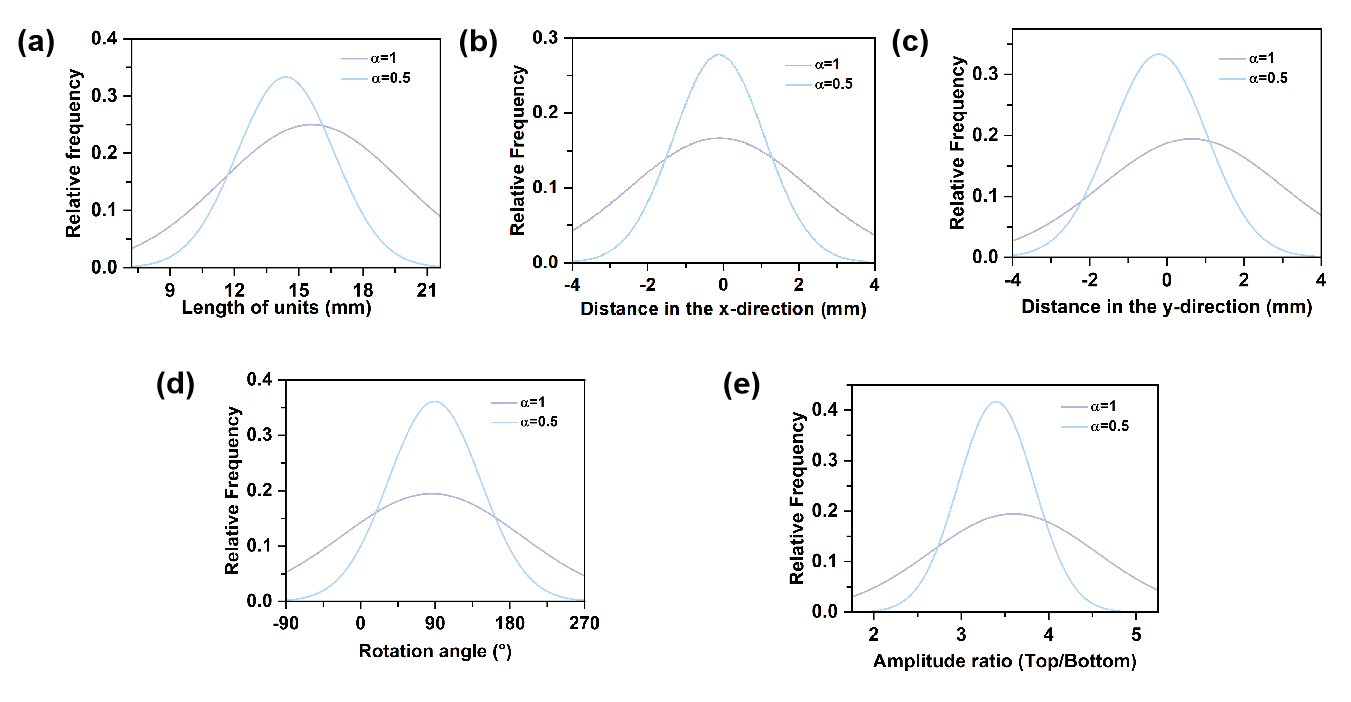


**Figure** **S3. Structural-feature distributions in disordered BCS (*α* = 0.5, 1).** Relative-frequency histograms for (a) unit length, (b) nodal spacing in x, (c) nodal spacing in y, (d) wall rotation angle and (e) amplitude ratio; broader, flatter curves confirm enhanced geometric randomness.


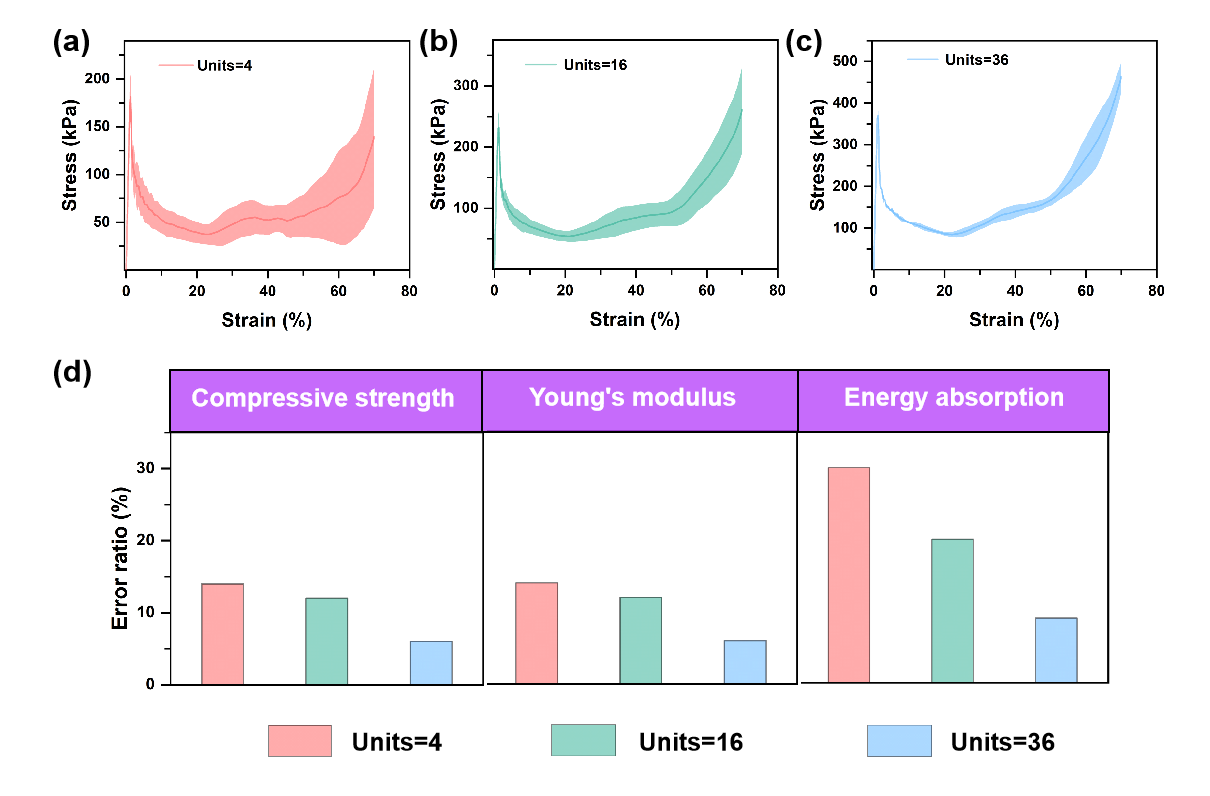


**Figure S4. Convergence study for fully disordered BCS (*α* = 1)** Stress–strain curves (mean ± SD) for lattices containing (a) 4, (b) 16 and (c) 36 units; scatter shrinks as unit count increases. (d) Coefficient of variation for compressive strength, Young’s modulus and energy absorption falls below 10% at 36 units, confirming representative-volume-element size.


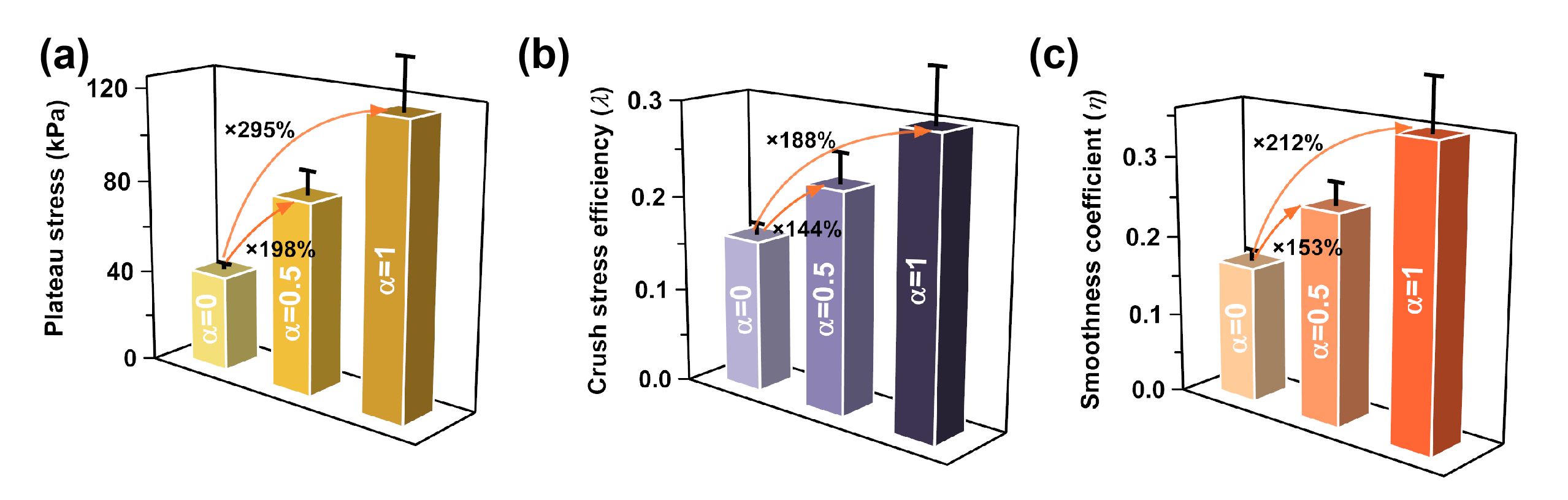


**Figure** **S5. Plateau metrics versus disorder.** (a) Plateau stress, (b) crush-force efficiency *λ* and (c) smoothness coefficient *η* of BCS (*α* = 0, 0.5, 1); all three indices rise monotonically with *α*, confirming that randomness stabilises post-yield crushing.


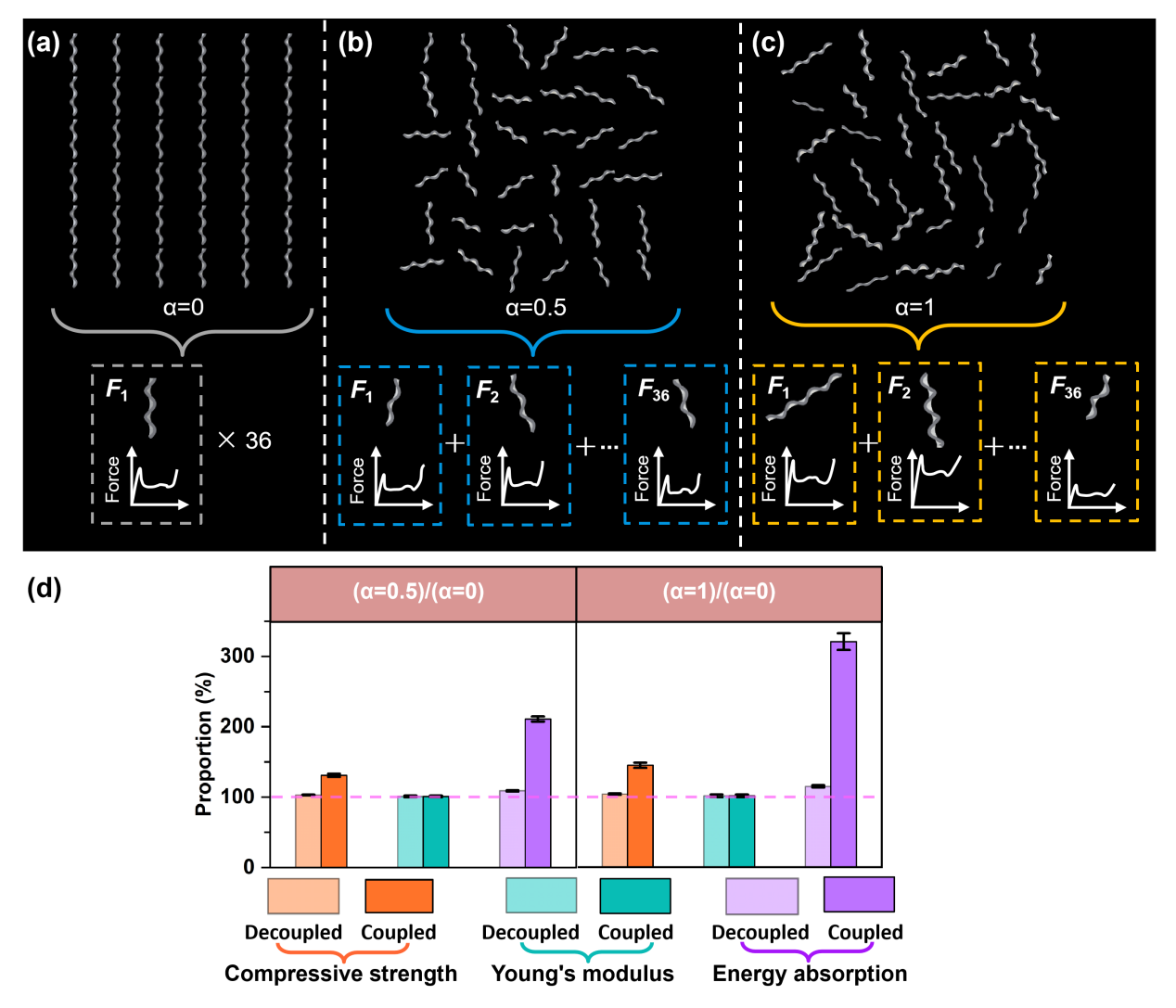


**Figure** **S6. Decoupled-unit superposition analysis.** (a–c) Schematic sequence showing force–displacement curves of individual cells linearly summed for *α* = 0, 0.5 and 1. (d) After removing geometric and density bias, decoupled disorder still raises strength and SEA by ≈ 41% and 206% versus the periodic BCS, whereas Young’s modulus is unaffected—confirming that randomness acts primarily through enhanced buckling resistance and contact dissipation.


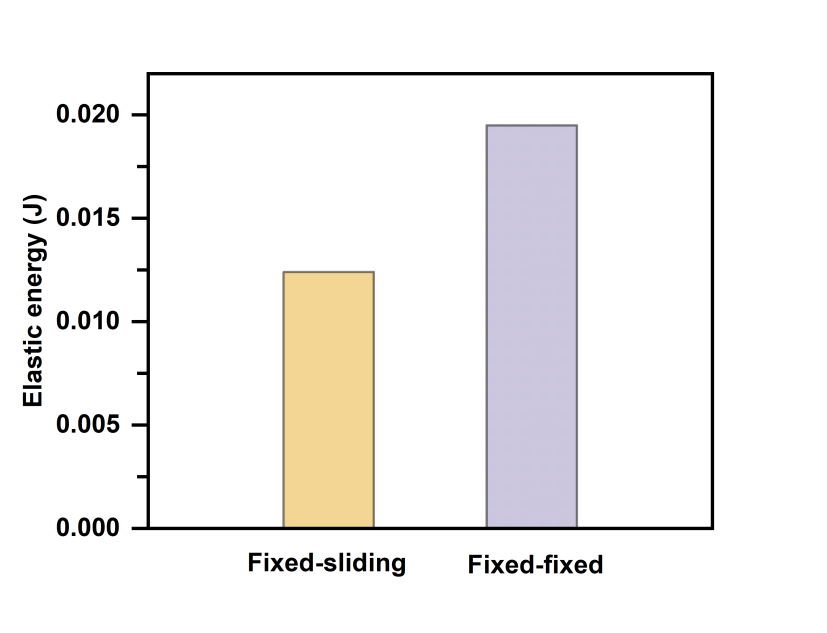


**Figure** **S7. Elastic energy of unit cells under fixed-sliding and fixed-fixed constraints.**


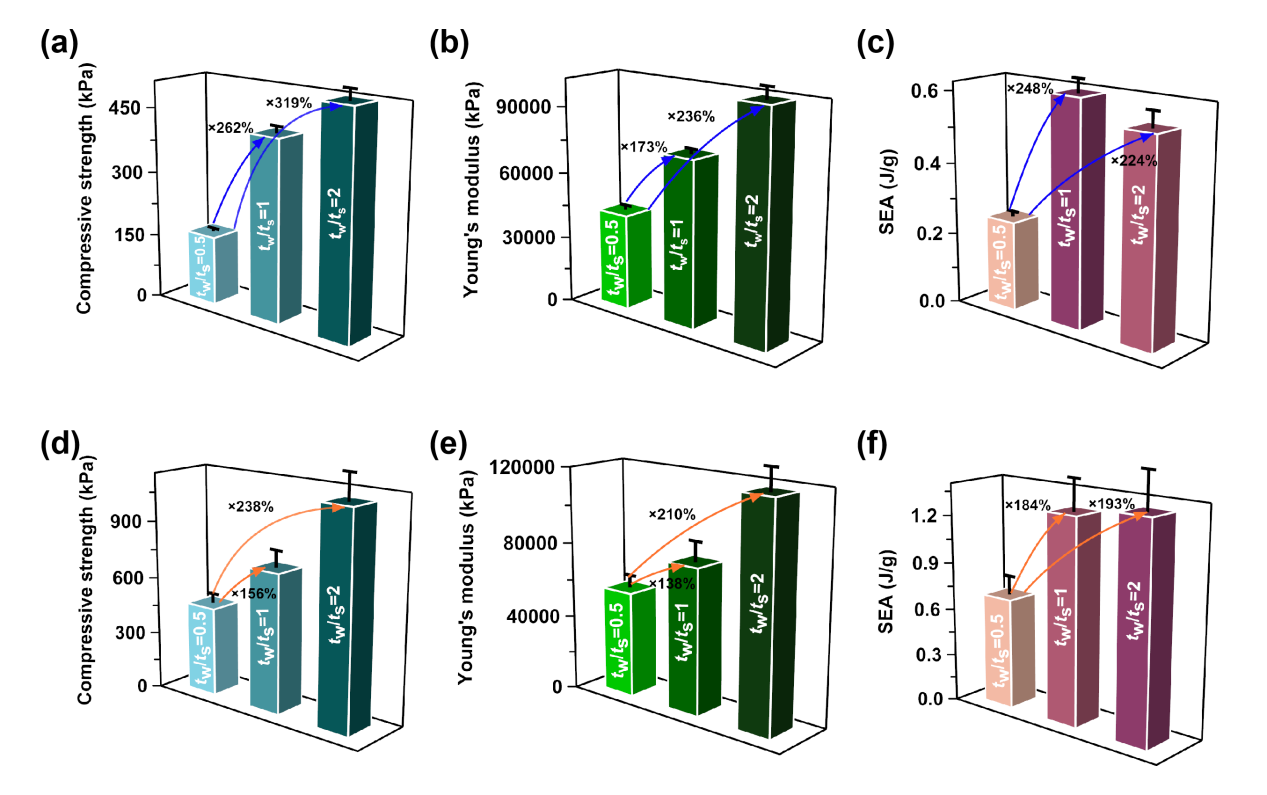


**Figure S8. Effect of wall-to-septum thickness ratio (*t*_w_/*t*_s_) on compressive response.** (a–c) Periodic BCS: strength and modulus rise with *t*_w_/*t*_s_, but SEA drops when the ratio increases from 1 → 2. (d–f) Fully disordered BCS: strength and modulus show identical trend, yet SEA plateaus (≤ 9% gain) from 1 → 2, demonstrating that appropriate selection of the ratio can further enhance the mechanical properties.

The custom shear fixture (Figure S9) consists of four key parts. Universal joints (components 1.1 and 2.1) automatically align the tensile axis with the specimen, eliminating parasitic bending from slight twist or clamp misalignment. Load transfer is secured by a hybrid bond-and-bolt scheme: steel bonding plates were sand-blasted and acetone-cleaned for maximum roughness and oil removal; high-strength epoxy was then brushed onto the plates and onto the sample (four 10 mm clearance holes around its perimeter); screws were inserted to register and pre-clamp the assembly; after a 24 h room-temperature cure the adhesive achieved full strength and the unit was ready for testing.


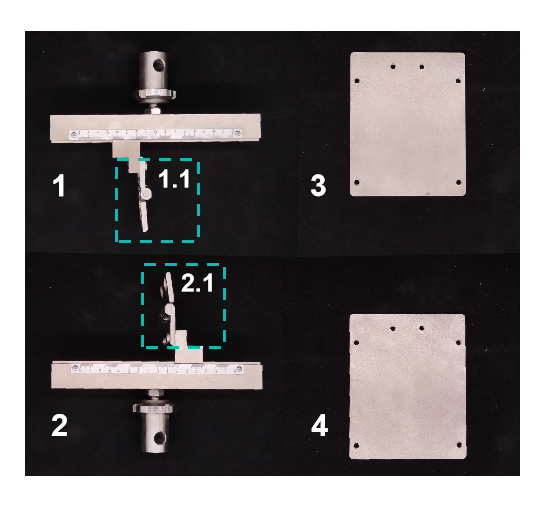


**Figure** **S9. Components of the shear fixture.**


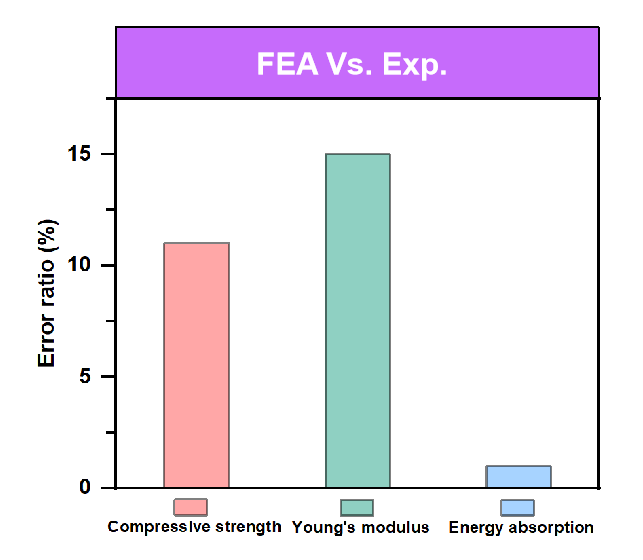


**Figure** **S10.** **Error ratio between finite element simulation and experimental results.**
